# SMRT sequencing of the full-length transcriptome of *Gekko gecko*

**DOI:** 10.1371/journal.pone.0264499

**Published:** 2022-02-25

**Authors:** Jianping Jiang, Juan Huo, Yueyun Zhang, Yongli Xu, Chengjian Zhao, Jianhua Miao

**Affiliations:** Guangxi Botanical Garden of Medicinal Plants, Nanning, Guangxi, China; ICAR-Indian Institute of Wheat and Barley Research, INDIA

## Abstract

Tokay Gecko (*Gekko gecko*) is a rare and endangered medicinal animal in China. Its dry body has been used as an anti-asthmatic agent for two thousand years. To date, the genome and transcriptome of this species remain poorly understood. Here, we adopted single molecule real-time (SMRT) sequencing to obtain full-length transcriptome data and characterized the transcriptome structure. We identified 882,273 circular consensus (CCS) reads, including 746,317 full-length nonchimeric (FLNC) reads. The transcript cluster analysis revealed 212,964 consensus sequences, including 203,994 high-quality isoforms. In total, 111,372 of 117,888 transcripts were successfully annotated against eight databases (Nr, eggNOG, Swiss-Prot, GO, COG, KOG, Pfam and KEGG). Furthermore, 23,877 alternative splicing events, 169,128 simple sequence repeats (SSRs), 10,437 lncRNAs and 7,932 transcription factors were predicted across all transcripts. To our knowledge, this report is the first to document the *G*. *gecko* transcriptome using SMRT sequencing. The full-length transcript data might accelerate transcriptome research and lay the foundation for further research on *G*. *gecko*.

## Introduction

The Tokay gecko (*Gekko gecko*, Linnaeus, 1758) is prevalent in southern China and Southeast Asia (Northeastern India, Birma, Anam, *etc*.) [1]. Its dry body is one of the rarest traditional Chinese medicines and is widely used in many Chinese patent medicines, such as Gejie Dingchuan capsule and Gejie Dingchuan pill [2,3]. Over the past few decades, because of the increasing medicinal demand for *G*. *gecko*, as well as ecological and environmental deterioration and excessive hunting, *G*. *gecko* has been listed as a Class II protected species in China since 1989 [4]. Although it is a significant species with high value in research and medicinal applications, genome and transcriptome information are still lacking.

RNA sequencing (RNA-seq) has become a powerful approach for generating a vast majority of sequence data and cDNA sequences, which might provide new and comprehensive information for genetic research [5]. For decades, a substantial number of RNA-seq studies have been conducted to understand gene expression and molecular mechanisms, moreover, RNA-seq is particularly widely used for nonmodel species that lack a reference genome [6–9], it provides insights into mRNA splicing and gene expression and has been used to screen candidate genes; however, the gene structure and full-length sequence are limited [10,11]. In addition, the extent of alternative splicing (AS) and transcriptome diversity remain largely unknown due to its short read length [12]. Recently, the single molecule real-time (SMRT) sequencing technique revolutionized the limitation of short read sequences and fragmentation, and postsequencing assembly are not needed. Moreover, SMRT sequencing provides accurate full-length transcripts, and average sequence read that up to 50 kb have been reported [13,14]. Therefore, SMRT sequencing represents an effective tool that has been widely and successfully used to annotate and analyze full-length transcripts among mammals, marine animals, aquatic animals and insects [15], such as *Tachypleus tridentatus [16]*, *Pinctada fucata martensii* [12], *Sogatella furcifera* [17], and *Odontotermes formosanu* [18–20]. However, no studies have investigated on *G*. *gecko*.

In this study, SMRT sequencing was used to generate full-length transcripts of *G*. *gecko*. A subsequent analysis of the transcriptome annotation and structure was performed. The results will provide a valuable and comprehensive genetic resource for further in-depth studies of gene function and biological regulatory mechanisms in *G*. *gecko*.

## Materials and methods

### Ethics statement

All procedures were performed in compliance with guidelines of the ethics committee of Guangxi Botanical Garden of Medicinal Plants.

### Sample collection and RNA preparation

One female cultured adult Tokay sample was collected from Nanning Junhao Wildlife Technology Development Co., Ltd., Guangxi, China, and then housed in the wood case in the specially culture room with a 12:12 day-night light cycle and 70% humidity, it was fed with *ad libitum* access to water and ground beetles (*Eupolyphaga sinensis* Walker) daily prior to euthanasia. The living specimen received anesthetic drugs and administered via intraperitoneal injection with potassium chloride (KCl) solution. Then, ten tissues, including heart, kidney, liver, lung, skin, blood, muscle, stomach, ovary, and oviduct, were dissected, immediately frozen in liquid nitrogen, and then stored at −80°C.

Total RNA was extracted from each tissue using the RNAiso Plus Reagent Kit (Takara Biotechnology, Dalian, China) according to the manufacturer’s instructions and then treated with RNase-free DNase I (TianGen, Beijing, China) to remove genomic DNA. The integrity and concentration of RNA were assessed using the Agilent Bioanalyzer 2100 system (Agilent Technologies, California, USA) and the Qubit^®^ 2.0 Fluorometer (Life Technologies, Carlsbad, CA, USA), respectively. High-quality RNA samples with RIN values ≥ 7.0 were equally pooled into one mixed sample used to construct the cDNA library for PacBio sequencing.

### Library construction, SMRT sequencing and quality control

Total RNA was reverse transcribed into cDNAs using a SMARTer cDNA Synthesis Kit (Takara Clontech Biotech, Dalian, China) according to the manufacturer’s protocols. Then, large-scale PCR was performed to generate more double-stranded cDNA templates. AMPure beads were used for the size selection of PCR products. The purified products of 0.4*beads and 1*beads were then mixed in equal quantities. After size selection, the PacBio Template Prep Kit was used to generate SMRTbell™ libraries. Finally, the SMRTbell™ libraries were sequenced with the Pacific Sequel platform.

### SMRT sequencing data processing

Raw reads were processed into circular consensus (CCS) reads using PacBio SMRT analysis software v2.3.0 (http://www.pacb.com/products-andservices/analytical-software/smrt-analysis/) to remove low-quality polymerase reads using the threshold of a read length < 50 bp and read score < 0.75. Full-length nonchimeric (FLNC) transcripts were determined by searching for both the 5’ and 3’ cDNA primers and the poly A tail signal in CCS. Consensus isoforms and FL consensus sequences were then obtained using iterative clustering for error correction (ICE) clustering analysis of FLNC. Additionally, high-quality FL transcripts were acquired by removing redundant sequences using CD-HIT (identity > 0.99) [21].

### Structure analysis and lncRNA prediction

MIcroSAtellite (MISA) software (http://pgrc.ipk-gatersleben.de/misa/) was applied to detect simple sequence repeats (SSRs) in the transcriptome. The noncoding DNA sequences within transcript sequences were predicted using TransDecoder (https://github.com/TransDecoder/TransDecoder/releases). Transcription factors (TFs) were identified based on the animalTFDB 2.0 database [22]. For AS event prediction, Iso-Seq^TM^ data were processed using all-vs.-all BLAST based on high identity settings [23]. Candidate lncRNAs were screened with the threshold of transcripts with lengths > 200 nt and more than two exons by combining the Coding Potential Assessment Tool (CPAT) [24], Coding-Non-Coding Index (CNCI) [25], Coding Potential Calculator (CPC) [26], and Pfam protein structure domain analysis (Pfam) [27].

### Functional annotation

All nonredundant transcript sequences were mapped to the following databases: National Center for Biotechnology Information (NCBI) nonredundant protein sequence database (Nr), Swiss-Prot database, Kyoto Encyclopedia of Genes and Genomes (KEGG), KOG/COG/eggNOG (Clusters of Orthologous Groups of proteins), Protein family (Pfam) and Gene Ontology (GO).

## Results

### Full-length transcript data output

First, 1–6 kb libraries were constructed based on the pooled RNA from ten tissues to perform PacBio SMRT sequencing and generate a comprehensive transcriptome for *G*. *gecko*. The analysis of transcriptome completeness with BUSCO showed that 67.7% (1,752 genes) were complete duplicated BUSCOs, 24.9% (645 genes) were complete single-copy BUSCOs, 2.4% (63 genes) were fragmented BUSCO archetypes, and 5.0% (126 genes) were missing BUSCOs (Table 1).

**Table 1:** Description of the BUSCO analysis.

BUSCO results	Count	Percentage (%)
Complete BUSCOs (C)	2,397	92.60%
Complete and single-copy BUSCOs (S)	645	24.90%
Complete and duplicated BUSCOs (D)	1,752	67.70%
Fragmented BUSCOs (F)	63	2.40%
Missing BUSCOs (M)	126	5.00%
Total BUSCO groups searched	2,586	

**Fig 1:**
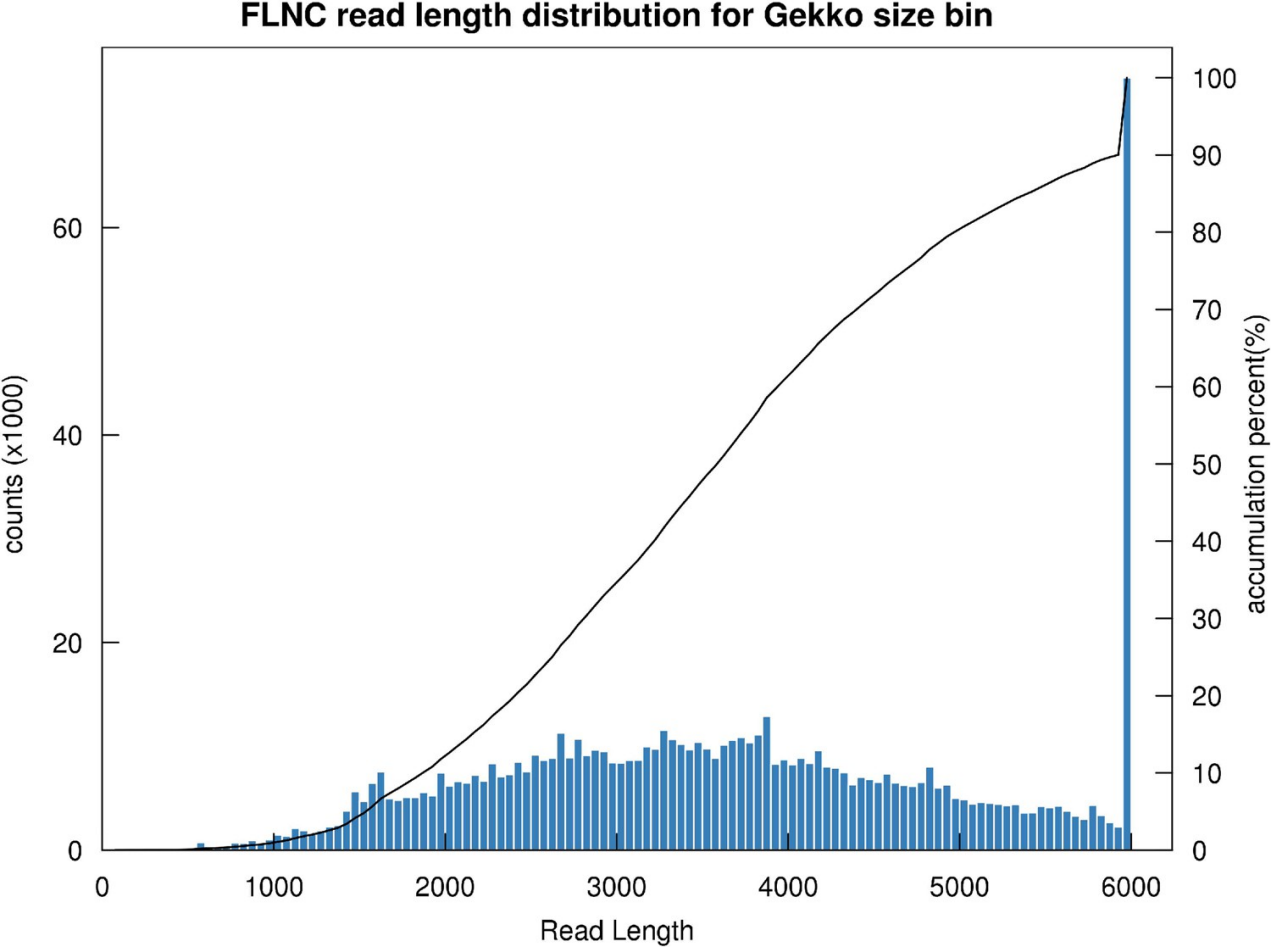
Distribution of the FLNC read length.

In total, 3.43 Gb of sequence data were obtained. A total of 882,273 circular consensus sequences were acquired with a mean length of 3,888 bp (Table 2). The subsequent analysis revealed 746,317 FLNC reads (Fig 1). After clustering, 212,964 consensus isoforms were generated with an average read length of 4,153 bp, resulting in 203,994 polished high-quality isoforms and 7,917 polished low-quality isoforms (Table 2). Finally, 117,888 nonredundant transcripts were generated.

**Table 2:** Summary of PacBio SMRT sequencing of *Gekko gecko*.

Category	Dataset
Read bases of CCS	3,430,277,475
Number of CCS	882,273
Mean Read Length of CCS	3,888
Number of undesired primer reads	79,372
Number of filtered short reads	0
Number of full-length nonchimeric reads	746,317
Full-length nonchimeric percentage (FLNC%)	84.59%
Number of consensus isoforms	212,964
Average consensus isoforms read length	4,153
Number of polished high-quality isoforms	203,994
Number of polished low-quality isoforms	7,917

### Functional annotation of transcripts

**Fig 2:**
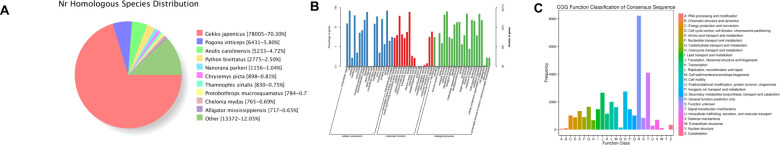
**(A)** The species identified by a homology search against the Nr databases. (B) GO annotation and (C) COG annotation of the *G*. *gecko* transcriptome.

In total, 111,372 identified transcripts were scanned against eight databases (S1 Table). The annotation rates were 111,001 (99.67%) in Nr, 109,042 (97.91%) in eggNOG, 91,887 (82.50%) in Pfam, 84,713 in GO (76.06%), 83,361 in KOG (74.85%), 75,001 in KEGG (67.34%), 73,152 in Swiss-Prot (65.68%) and 34,491 in COG (30.97%) (Table 3). Based on the Nr annotation, the prediction of species homologous with *G*. *gecko* was performed via sequence alignments. Consequently, *Gekko japonicas* showed a close evolutionary relationship with *G*. *gecko* (Fig 2A).

**Table 3:** Statistics of the annotation results.

Annotated databases	Isoform number	Percentage
Nr	111,001	99.67%
eggNOG	109,042	97.91%
Pfam	91,887	82.50%
GO	84,713	76.06%
KOG	83,361	74.85%
KEGG	75,001	67.34%
Swiss-Prot	73,152	65.68%
COG	34,491	30.97%
All database	111,372	94.47%

GO enrichment analysis was performed to classify the functions of all full-length transcripts (Fig 2B). The results revealed that 84,713 transcripts were classified into three main categories: cellular component (CC), molecular function (MF) and biological process (BP). In the three categories, cellular process (54,599 transcripts), single-organism process (42,048 transcripts) and cell part (60,809 transcripts) were the main terms identified in BP, MF and CC, respectively. COG classification was also performed to further study the functions of the *G*. *gecko* transcripts. The COG analysis showed that 34,491 transcripts were grouped into 24 categories. The dominant subcategory was general function prediction only (8,220, 23.83%), followed by signal transduction mechanisms (4,111, 11.92%) and posttranslational modification, protein turnover, and chaperones (4,722, 7.99%) (Fig 2C).

KEGG pathway analysis was conducted to understand the biological function of the *G*. *gecko* transcriptome. The results showed that 75,001 (67.34%) transcripts were enriched in 303 signaling pathways. Among them, endocytosis (2,464, 3.29%) and focal adhesion (1,564, 2.09%) were the major pathways, followed by the MAPK signaling pathway (1,522, 2.03%), regulation of actin cytoskeleton (1,497, 2.00%), and tight junction (1,466, 1.95%) (Table 4).

**Table 4:** The top 20 mapped pathways annotated by the KEGG database.

Pathways	Pathway ID	Gene number	Percentage
Endocytosis	ko04144	2,464	3.29%
Focal adhesion	ko04510	1,564	2.09%
MAPK signaling pathway	ko04010	1,522	2.03%
Regulation of actin cytoskeleton	ko04810	1,497	2.00%
Tight junction	ko04530	1,466	1.95%
Herpes simplex infection	ko05168	1,431	1.91%
Protein processing in endoplasmic reticulum	ko04141	1,307	1.74%
Phagosome	ko04145	1,239	1.65%
RNA transport	ko03013	1,199	1.60%
Purine metabolism	ko00230	1,167	1.56%
Insulin signaling pathway	ko04910	1,116	1.49%
Ubiquitin mediated proteolysis	ko04120	1,104	1.47%
mTOR signaling pathway	ko04150	1,076	1.43%
Spliceosome	ko03040	1,064	1.42%
Calcium signaling pathway	ko04020	1,052	1.40%
FoxO signaling pathway	ko04068	1,013	1.35%
Apoptosis	ko04210	984	1.31%
Lysosome	ko04142	979	1.31%
Adherens junction	ko04520	946	1.26%
Adrenergic signaling in cardiomyocytes	ko04261	941	1.25%

### SSR detection

A total of 169,128 SSRs were identified in 72,630 SSR-containing sequences using the MISA tool. Among these transcripts, 42,163 contained more than one SSR. Furthermore, the most abundant was mononucleotides (104,516, 61.80%), followed by dinucleotides (33,648, 19.89%). The frequencies of tri-, tetra-, penta- and hexanucleotides were 15.51% (26,224), 2.45% (4,137), 0.29% (488), and 0.07% (115), respectively (Table 5). All SSRs and the corresponding primers are listed in S2 Table.

**Table 5:** Statistical analysis of SSRs.

Item	Number
Total number of sequences examined	116,842
Total number of sequences examined (bp)	517,279,084
Total number of identified SSRs	169,128
Number of SSR-containing sequences	72,630
Number of sequences containing more than 1 SSR	42,163
Mononucleotides	104,516
Dinucleotides	33,648
Trinucleotides	26,224
Tetranucleotides	4,137
Pentanucleotides	488
Hexanucleotides	115

### LncRNA prediction

**Fig 3:**
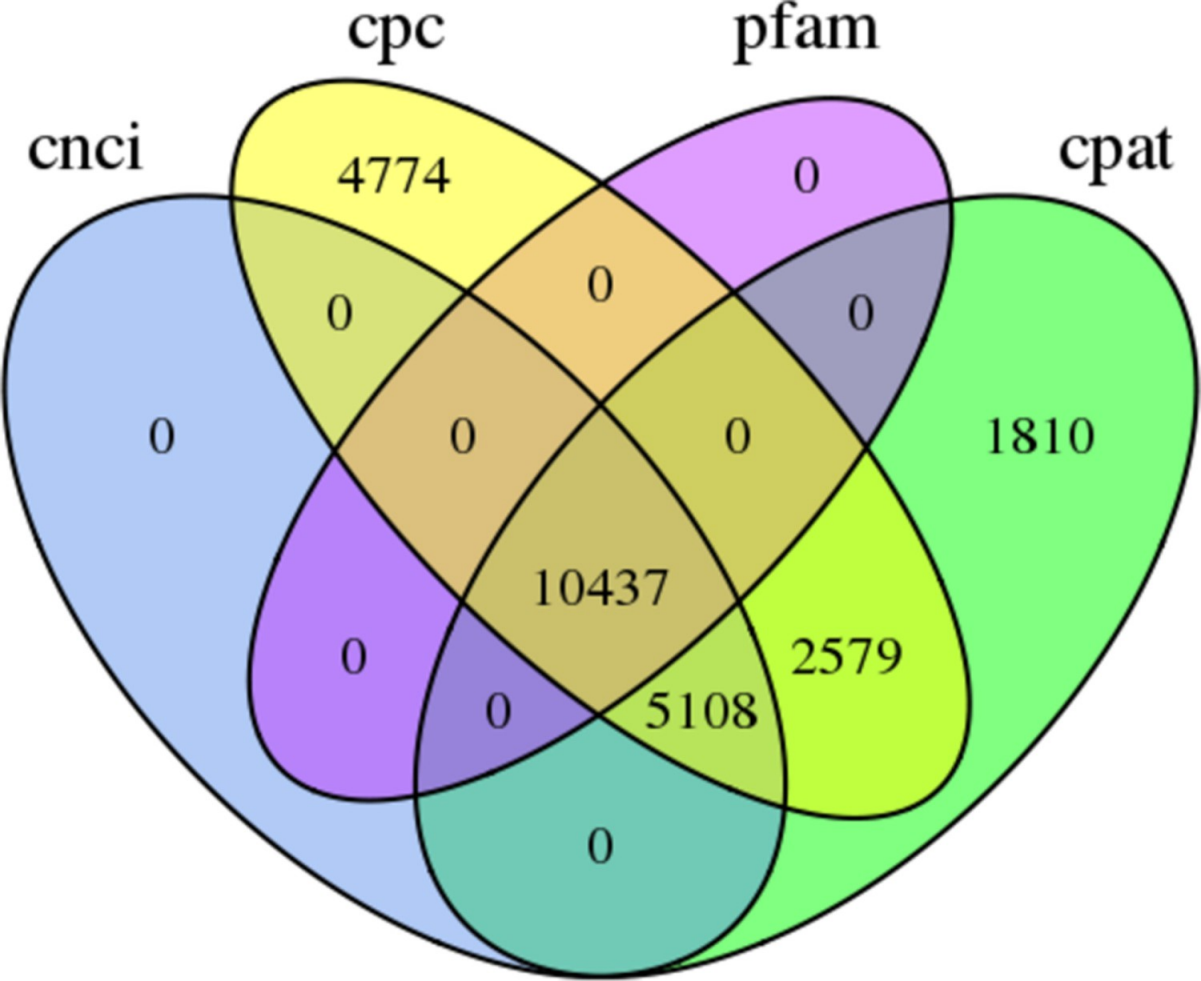
Candidate lncRNAs identified by CPC, CNCI, CPAT and Pfam.

Four computational tools were combined and used to predict lncRNAs, including the CPC, CNCI, CPAT and Pfam databases. The results revealed that 22,898, 15,545, 19,934, and 10,437 lncRNAs were obtained from the CPC, CNCI, CPAT and Pfam databases, respectively. Among them, 10,437 lncRNAs were identified by the four approaches (Fig 3).

### Prediction of ORFs, AS and TFs

**Fig 4:**
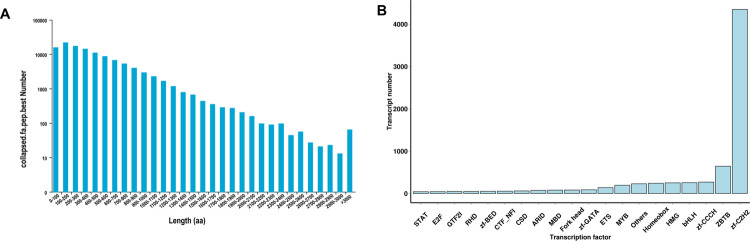
**(A)** Length distribution of CDSs and (B) type distribution of TFs.

In total, 91,948 ORFs were identified using TransDecoder v3.0.1 software. As shown in Fig 4A, CDSs ranging from 100 bp to 200 bp were dominant (21,919, 18.75%). A total of 23,877 alternatively spliced sequences were defined (S3 Table). Furthermore, 7,932 TFs were detected using the animalTFDB 2.0 database, of which the major types were members of the ZBTB and zf-C2H2 families (Fig 4B).

## Discussion

Based on accumulating evidence, the dry body of *G*. *gecko* exerts remarkable effects on strengthening the immune system and treating tumors [28–30]. As an economically important Chinese medicinal animal, obtaining a full-length transcriptome and understanding the structure of genes in *G*. *gecko* is a primary step in studying gene function, which is very important, yet it is still unknown.

SMRT sequencing provides new knowledge of full-length sequences, which is confirmed to be useful for performing gene annotation and interpreting gene functions, especially for species lacking a reference genome [12,31]. In the present study, we obtained 882,273 CCSs, identified 746,317 FLNC, and then yielded 212,964 corrected isoforms with an average read length of 4,153 bp. Compared with short-read sequencing (e.g., Illumina sequencing), the mean length of SMRT-sequenced transcripts was greater than 3 kb, which far exceeded the value reported in previous studies analyzing *Heloderma horridum horridum* [32], *Gekko japonicas* [33], *Palaemon serratus* [34], and *Henosepilachna vigintioctopunctata* [35]. Furthermore, 117,888 high-quality unique full-length transcripts were generated based on the high competence of PacBio SMRT sequencing, and 111,372 transcripts were successfully annotated with 116,913 ORFs. To our knowledge, this study is the first to characterize the full-length transcriptome of *G*. *gecko*, and the results might substantially accelerate further research.

Here, the percentage of annotated transcripts was 94.47%. GO and COG classifications revealed that major transcripts were involved in cellular process, single-organism process, biological regulation, metabolic process, signal transduction mechanisms, posttranslational modification, protein turnover, chaperones, translation, and ribosomal structure and biogenesis. Notably, 2,464, 1,564, and 1,522 transcripts were involved in endocytosis, focal adhesion, and the MAPK signaling pathway, respectively.

Alternative splicing and transcription factors are involved in transcriptional mechanisms that regulate gene expression [35,36]. We identified 23,877 AS events and 7,932 TFs in *G*. *gecko*. lncRNAs are defined as nonprotein-encoding transcripts with a length of more than 200 nucleotides [37–39]. Researchers have now appreciated that lncRNAs function as local regulators to mediate the expression of neighboring genes through RNA–protein interactions [39–41]. However, no lncRNAs have previously been reported in *G*. *gecko*. In our study, 10,437 common lncRNAs were predicted by four software programs, which will promote further functional research of these lncRNAs in the *G*. *gecko* transcriptome.

## Conclusion

We acquired a high-quality *G*. *gecko* transcriptome using the PacBio SMRT sequencing platform. The results are very valuable to facilitate the future annotation of the *G*. *gecko* genome and optimize the gene structure. Furthermore, the findings may provide important information for research on gene functions in this species in the future.

## Supporting information

S1 Table Functional annotation of identified transcripts. (XLS)

S2 Table Predicted SSRs. (XLS)

S3 Table Identified alternative splicing sequences. (XLS)
